# Hybrid Learning Models for IMU-Based HAR with Feature Analysis and Data Correction

**DOI:** 10.3390/s23187802

**Published:** 2023-09-11

**Authors:** Yu-Hsuan Tseng, Chih-Yu Wen

**Affiliations:** 1 Department of Computer Science and Engineering, National Chung Hsing University, Taichung 40227, Taiwan; g110056067@mail.nchu.edu.tw; 2 Department of Electrical Engineering, National Chung Hsing University, Taichung 40227, Taiwan; 3 Smart Sustainable New Agriculture Research Center (SMARTer), National Chung Hsing University, Taichung 40227, Taiwan; 4 Innovation and Development Center of Sustainable Agriculture (IDCSA), National Chung Hsing University, Taichung 40227, Taiwan

**Keywords:** human activity recognition, variational autoencoder, generative adversarial networks

## Abstract

This paper proposes a novel approach to tackle the human activity recognition (HAR) problem. Four classes of body movement datasets, namely stand-up, sit-down, run, and walk, are applied to perform HAR. Instead of using vision-based solutions, we address the HAR challenge by implementing a real-time HAR system architecture with a wearable inertial measurement unit (IMU) sensor, which aims to achieve networked sensing and data sampling of human activity, data pre-processing and feature analysis, data generation and correction, and activity classification using hybrid learning models. Referring to the experimental results, the proposed system selects the pre-trained eXtreme Gradient Boosting (XGBoost) model and the Convolutional Variational Autoencoder (CVAE) model as the classifier and generator, respectively, with 96.03% classification accuracy.

## 1. Introduction

In recent years, artificial intelligence and machine learning technologies have gradually matured, and smart environments have flourished. In particular, human attitude recognition (HAR) based on wireless sensor networks is widely used in the fields of smart homes, medical applications, national security, driving activity recognition and smart cars [1], elderly home monitoring and rehabilitation [2,3], and industrial tasks related to human–robot interaction [4], which have significantly improved people’s quality of life.

HAR can identify and capture human body movements or posture through various sensors. In early systems, the Microsoft Kinect camera was used to collect RGB, grayscale, and depth images of the human body, which were fed as input data to perform image recognition to complete gesture recognition and fall detection [5]. However, under the fixed camera lens, RGB images can only work in well-lit spaces with a limited field of view and may lead to privacy violations. Therefore, nowadays, the non-vision-based HAR is an important research topic. Our concept is to wear various types of sensors on the human body and analyze the movement and posture changes in the human body by measuring the dynamic parameters of the limbs. Common devices include accelerometers, gyroscopes, and infrared and ultrasonic sensors [6]. Among them, the inertial measurement unit (IMU) sensor is chosen due to its high sampling rate, rapid detection of inertial parameters (e.g., linear acceleration, angular acceleration, and quaternion of the limb), low power consumption, and high precision [7]. It is widely used in the field of HAR to form a body area network (BAN) [8] to identify the whole-body movement.

Since IMU signals are utilized to track body activity and gestures, not too much privacy will be revealed, and users will not be limited by the activity scope when performing daily activities. However, the sensor measurements with wearable sensors deployed on the body are subject to signal interference due to the changing positions and noise issues, leading to severe measurement drift and resulting in poor accuracy. Therefore, Mahony [9] proposes a non-linear complementary filter based on proportional–integral–derivative error compensation control, which reduces the error by 99%, making it comparable to the result of the Euler angles without using the filter. Moreover, Madgwick [10] uses the gradient descent algorithm to derive the quaternion while minimizing the error. To compensate the error caused by the complex motion noise, this work uses Madgwick Filter to obtain the attitude quaternions via the accelerometer and the gyroscope integration, which are then linearly fused to obtain the optimal attitude.

In general, the recognition of the body movement needs multiple sensor nodes to track the dynamic parameters of the specific joint points. Considering the inconvenience and the cost of the wearable devices, in this work, we propose a BAN system based on an IMU sensor node, which is deployed on the subject’s waist. The IMU node consists of an accelerometer, a gyroscope, and a magnetometer. Using the Madgwick filter, the noise and measurement drift are reduced to capture the correct inertial signal. Then, based on the Wi-Fi wireless technology, the data packet is transmitted to the database, which contains the inertial signal information. Four classes of body movement datasets, namely stand-up, sit-down, run, and walk, are applied to perform HAR.

In addition to using the time series of inertial signals as input features, we propose a new method to convert the time series of inertial signals into images with varying image sizes, as well as suppress the impact of the noise via feature extraction and feature selection. Moreover, we generate a fake database using several types of generative models to increase the amount of data and re-train the model, improving the classifier generalization such that the purpose of human activities classification with high precision can be achieved.

The main contributions and features of this study are as follows:

- In non-vision situations, one IMU sensor node is applied to distinguish the whole-body movement between motion transition and a continuous motion.
- We propose a method of generating images from time series data that visually indicate the characteristics of inertial signals and generate multiple fake images based on AE and GAN models to improve classification accuracy.
- We evaluate the accuracy and characteristics of classification models. The results show that XGBoost has the best results in non-uniform actions using a small amount of data.
- We evaluate the classification accuracy using fake images and select the best-accuracy to perform for data correction.
- We propose a real-time BAN system to correct inertial signal data and classify human body movements.

The organization of this paper is as follows: Section 2 reviews related works about inertial-sensor-based HAR approaches. Section 3 presents the proposed system architecture and discusses the proposed methodology, including data pre-processing, classification algorithms, correction algorithms, algorithm optimization, and evaluation methods. Section 4 evaluates the system performance of the classification and data correction, presents the performance comparison, and examines the real-time HAR system. Finally, Section 5 draws conclusions and outlines future research directions.

## 2. Related Works

With the development of sensor technology, wearable sensors are widely used in most electronic devices, such as smartphones, watches, and bracelets. These devices are usually mounted onto a part of the body, such as the wrists [11], arms [12], ankles [13], or waist [14]. They can directly capture data without any area restrictions, as well as analyze the body movement. In HAR systems, common wearable sensors include electromyography (EMG), electrocardiography (ECG), accelerometer, and IMU nodes. Among them, IMU sensors are the most widely used because of their light weight and acquisition of a variety of inertial signals.

HAR systems are usually based on the BAN concept, allowing users to wear sensors at multiple joint points [15,16,17] to facilitate feature extraction and action classification. However, the need to wear multiple sensors is likely to cause users to feel that sensors are intrusive [18]. Therefore, HAR systems with a single IMU have become a popular research topic recently. Although a single IMU can only capture limited information, its accuracy and cost can be greatly improved when the signals captured via its different modules are fully utilized [19]. The classification results using a mix of different modules versus a single module as the feature input are compared. The results show that using all signals captured via the nine-axis IMU as input features is leads to significantly higher accuracy than other combinations. Moreover, Kangas [20] evaluates the IMU performances with various wearing areas of the body. The classification performances at the waist and on the head are shown to be more accurate than that at the wrist.

Real-time HAR systems for use in low-power wireless communication are proposed [21,22,23]. The authors [21] deploy an IMU sensor at the waist to transmit its signal to a local personal computer (PC) via Zigbee wireless communication technology. The signal processing and classification algorithm are executed on the PC side to perform long-term dynamic monitoring of the family. The results show that it can distinguish between sitting, lying, and standing movements with high accuracy, but it has limited ability to detect slow walking. In [22], 2.4-gigahertz radio frequency communication is applied, which has the characteristics of low cost and plug and play, but it has the disadvantages of a weak signal, a short transmission distance, and an inability to immediately upload data to the cloud database. Accordingly, in order to remove distance constraints and improve convenience, in this work, Wi-Fi wireless networking technology is applied to upload data to the mySQL [24] cloud database in a timely manner.

In [25], the authors develop a wrist-worn node, consisting of an IMU sensor and a Wi-Fi chip, to perform athlete tracking, as the tracking data can be transmitted to the cloud database and then transmitted to the visual web page or APP through the MQTT protocol, allowing coaches to monitor the status of athletes and correct their wrong movements in real time. In contrast to [25], the system proposed in this work only transmits the device status to the user interface (UI) to provide monitoring information based on Wi-Fi communication and does not need to transmit inertial signals to the UI to perform calculation. We note that the user datagram protocol is used to improve the transmission speed.

In order to accurately identify the current human activity, the HAR system integrates the IMU signals with the concept of artificial intelligence and machine learning techniques (e.g., support vector machine (SVM) [19,26], K-nearest neighbor (KNN) [26], hidden Markov model (HMM) [27], decision tree (DT) [19], random forest (RF) [26] and AdaBoost [28]. In [29], a waist-worn HAR system is proposed via the DT classification algorithm, which classifies various human activities, including walking, falling, and resting. In [30], a hybrid classification method is developed to detect fall events in older people, which uses principal component analysis (PCA) to standardize and reduce the dimension of the data and reduce unimportant features. The processed features are then fed into machine learning models, such as SVM and KNN, to complete action classification. In [31], time domain characteristics, such as mean absolute value (MAV), variance (VR), and root mean square (RMS), are captured to obtain the time series information of IMU sensors. The correlation-based feature selection (CFS) approach is used to eliminate redundant features. It is then fed into machine learning models, such as KNN and RF. The results show that the classification method based on CFS and RF has the best accuracy. Accordingly, feature extraction and feature selection are important for HAR.

Recent studies [32,33,34] show that the use of deep learning technology to study human pose recognition has become an important research trend. Deep learning networks have powerful non-linear representation learning capabilities and can automatically capture data features required for the classification of complex actions [35]. The authors [36] feed the characteristics of the time domain of inertial signals into nine different neural network models to complete the action classification and prediction of subjects. The model is based on CNN, RNN, and convolutional recurrent neural network (CRNN) architectures. After five cross-verifications, the results show that this hybrid deep learning method is superior to traditional machine learning algorithms, such as KNN and DT. In [37,38], the CNN method is used to screen the features of time series data and eliminate the features of the data under a specific topic to improve the generalization of the model.

Compared to traditional machine learning algorithms, although deep learning methods result in better classification accuracy, the model’s complexity is high, the number of model parameters is large, and the classification results cannot be reflected in time. In addition, neural networks often require a large amount of data to ensure the validity of model accuracy. The authors [39] compare the accuracy of traditional machine learning algorithms (e.g., SVM, KNN, and RF) to deep learning algorithms (e.g., CNN and LSTM) at different scales regarding the number of datasets. The results show that datasets with a small amount of data are suitable for performing traditional machine learning. Conversely, datasets with large data volumes are suitable for using deep learning to complete action classification.

A sufficient amount of training data is one of the key elements required to improve the HAR accuracy. However, in practical applications, it may be difficult to obtain sufficient training data. The HAR system developed by the authors [40,41,42] shows that due to its limited ability to capture information, a single IMU can only recognize continuous actions and cannot identify actions containing switching processes, despite the good feature processing and classification methods used. The authors in [40] classify the body actions into four categories, considering continuous actions with a classification accuracy 98.88%. However, the transition process (i.e., from the current action to the new action) is not considered. The gesture recognition system in [41] completes 14 specified gestures in a specified time and uses mRMR technology. In [42], the recognition of the movement between the gesture and the arm is completed within a specified time, and the sequence sensing information of the IMU is filtered via the EMG Sensor to ensure that the motion observation is maintained in a continuous motion status. To overcome the above problems, we propose a data augmentation strategy for actions that include switching action processes and develop generative models based on an autoencoder (AE) and a generative adversarial network (GAN). By learning the hidden features of the data derived from the original distribution, new data are generated under the same distribution [43]. The authors [44,45,46,47,48,49,50] propose a variety of GAN models to generate realistic sensor data, thereby improving the accuracy and generalization of the model. Table 1 summarizes various data augmentation strategies based on generative models.

In existing HAR systems, features are often independently extracted from multiple time series sensor signals in a hand-crafted manner. Correlations between different signals are often ignored [14]. Therefore, in this work, we suggest that all inertial signal sequences from accelerometers, gyroscopes, and magnetometers can be represented by a new active image, which contains the hidden relationship between the signals. Moreover, the studies [14,51,52] show that the newly generated image can provide additional contextual information regarding the signal compared to the statistics of a single time series signal and has better classification results in algorithms such as machine learning or deep learning methods.

## 3. System Description

**Figure 1:**
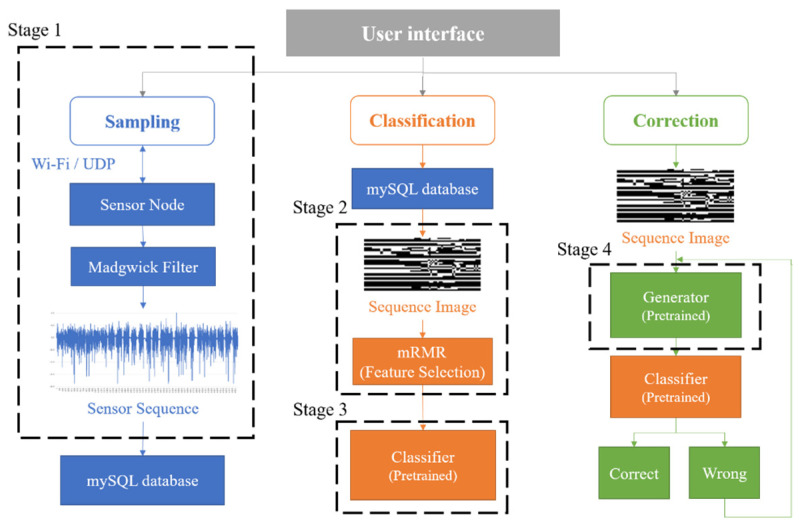
The proposed system architecture based on real-time HAR.

Figure 1 shows the proposed real-time HAR system architecture, which aims to achieve the data sampling of human activity, data pre-processing, real-time activity classification, data generation, and sensory correction by providing a simple UI for the users. The system implementation comprises the following four stages:

- Stage 1—Networked sensing and data sampling: The data collection process is performed via a BAN system based on Wi-Fi wireless technology.
- Stage 2—Data pre-processing: A new image generation method is developed to preserve the correlation between each inertial signal and filter features via the PCA and the minimum redundancy–maximum relevance (mRMR) feature selection algorithm. Moreover, images at various scales are generated to determine the appropriate image sizes.
- Stage 3—Activity classification: We determine effective models by integrating algorithms (e.g., traditional machine learning, deep neural network, and transfer learning) and hyperparameter optimization methods (e.g., K-Fold cross-validation, GS, and RS).
- Stage 4—Data generation: Multiple generative models are developed to implement data augmentation and improve data generalization. Then, we select the pre-trained generative model with the highest accuracy to execute data correction.

### 3.1. Stage 1: Networked Sensing and Data Sampling

This section describes the sensor node used in the developed BAN system, which consists of a nine-axis IMU and a NodeMCU-32S Wi-Fi microcontroller. To determine the desired nine-axis IMU, we compare the specifications of the two most common nine-axis IMUs, MPU9150 (from InvenSense-TDK Corporation [53]), and BNO055 (from Bosch Sensortec [54], Kusterdingen, Germany), and we consolidate the results in Table 2. Referring to the table below and considering the importance of sensor accuracy (such as accelerometer and gyroscope sensors) to this system, the MPU9150 is used as the nine-axis IMU for the target output in this work.

Moreover, we choose the NodeMCU-32S microcontroller as the central processing unit, which is responsible for controlling the peripheral sensors (e.g., controlling the RGB LED light signal to display the node status and transmitting the acquisition command to control the nine-axis IMU). We note that the sensors are calibrated each time when measuring a new action. The MPU9150′s Register Map document [55] is applied to correct the gyroscope sensor and an accelerometer sensor. The magnetometer data are corrected with reference to MPU9150′s datasheet [56].

**Figure 2:**
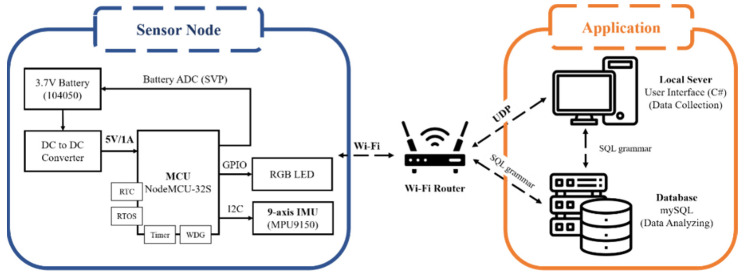
System structure of the proposed wearable sensor node.

**Figure 3:**
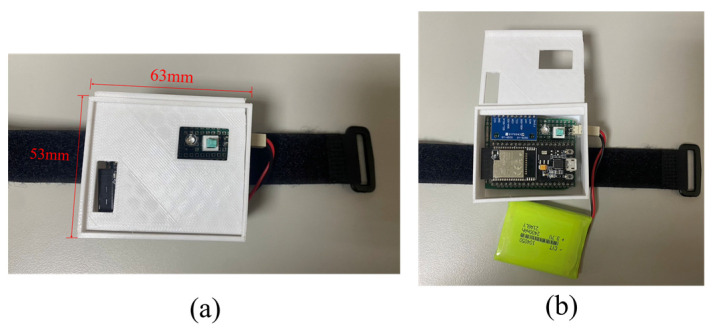
(**a**) The size of the proposed wearable sensor node; (**b**) the top view of the sensor node.

Figure 2 and Figure 3 show the system structure and hardware components of the sensor node, respectively. The nine-axis IMU is composed of a three-axis gyroscope, three-axis accelerometer, and three-axis magnetometer, which capture three-axis angular velocity, three-axis acceleration, and three-axis magnetic field signals, respectively. The inertial signals are further analyzed to obtain movement information (e.g., Euler angle, quaternion, and three-axis linear–angular velocity). Subsequently, the Madgwick gradient descent attitude algorithm is applied to minimize the quaternion error and gyroscope drift, thereby providing accurate attitude estimation. In the process of algorithm implementation, the gyroscope data are used to update the quaternion initially. Then, the acceleration data and magnetometer data are calculated and processed based on the gradient descent method to find the quaternion components with the minimum error. Finally, the two approaches are fused together to obtain the final pose quaternion. The equation for attitude estimation with gradient descent is

(1) $$q_{t}=q_{t-1}+\left({∆}_{gyr}-\beta \cdot S\right)\cdot dt,$$

where $q_{t-1}$ is the previous orientation, ${∆}_{gyr}$ is the quaternion change rate calculated from the gyroscope, β is the optimal steepness of the gradient descent, and S is the corrective step quaternion calculated based on its current direction and accelerometer data when using the gradient descent method.

**Figure 4:**
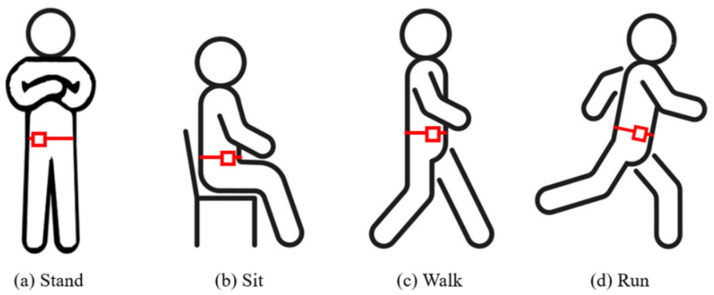
The activities involved in this study, with each red square representing the corresponding location of the wearable sensor node used to track each activity.

**Figure 5:**
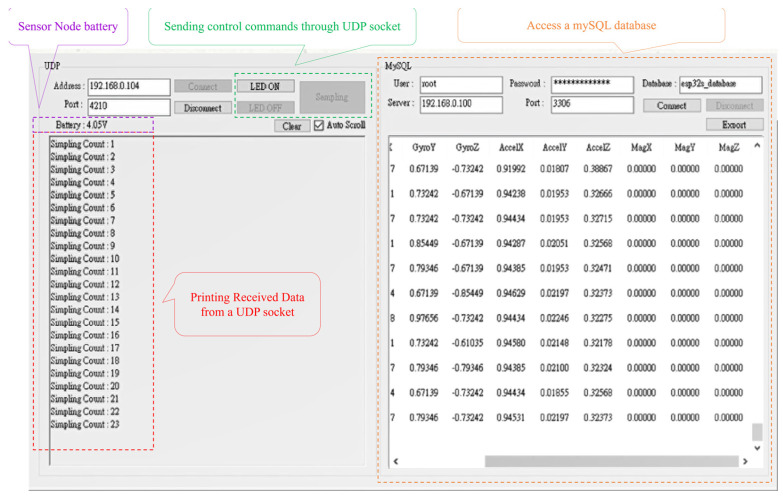
User interface of the proposed wearable sensor node.

With regard to data collection, the waist-worn sensor node collects the sensing measurements of each action at a sampling rate of 10 Hz and during windows of 10 s, including standing, sitting, walking, and running activities, as shown in Figure 4. Boerema et al. [57] discuss the results of the full-body movement of IMU devices worn on various parts of the waist and investigate the optimal sensor placement used to measure physical activity. The results show that the most preferred transverse locations on the belt are the following two locations: the right hip anterior position (position 1) and the right hip most lateral position (position 2). Moreover, the exploration of how closely the sensors fit with the body suggests that to achieve the best results, the sensors should be mounted as closely as possible on the body. This tight fit can be promoted by providing mounting materials that ensure this tight fit, such as an elastic belt or a clip that has a strong connection with the belt. Therefore, with reference to [57], in this work, the sensors are located between positions 1 and 2 to facilitate wearing and ensure that the user can move freely. Moreover, we design a simple visual UI (Figure 5) using the C sharp programming language. This system allows the system to monitor the node status in a user-friendly manner and quickly access the data in the mySQL cloud database.

### 3.2. Stage 2: Data Pre-Processing

**Figure 6:**
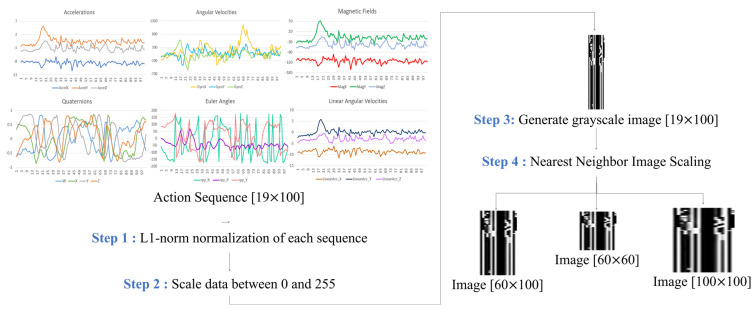
Flowchart of the conversion of a sequence into an image.

Instead of directly applying multiple inertial signal sequences as a feature, this work proposes a novel method that converts the signal sequences into new images, which can effectively express the correlation between signals [28,29,30]. Firstly, we perform the progressive L1 norm normalization of the captured action sequence data using a 19 × 100 matrix and reduce the value range of each column to [0, 1], with the sensing data being collected from the 19 parameters (i.e., three-axis angular velocity, three-axis acceleration, three-axis magnetic field, Euler angles (a set of three angles), and quaternions (containing four components), and three-axis linear angular velocities), and the action sequence of each individual parameter is represented by a 1 × 100 raw vector. We note that 100 represents the number of sampling points captured by each action at a sampling rate of 10 Hz during observation windows of 10 s. Next, we enlarge the normalized value to fit the range [0, 255] and convert it into a single-channel grayscale image with an image size of 19 × 100. Finally, we generate images of different sizes (e.g., 60 × 100, 60 × 60, and 100 × 100) based on nearest neighbor interpolation to improve the resolution, allowing us to find the most appropriate image generation method. Figure 6 describes a flowchart used to convert sequences into images.

The increased number of data features leads to an increase in the dimensionality of the data, which may lead to model overfitting and take a long training time to occur due to a large amount of calculation. The most common approaches of feature engineering include feature extraction (FE) and feature selection (FS). FE converts the original features through equations, thereby reducing the input feature dimension. FS is used to find the optimal feature subset by screening the existing features and removing irrelevant or redundant features. Therefore, this study proposes using the PCA and mRMR analyses to complete feature engineering.

In the original feature set, mRMR uses F-statistic to calculate the correlation between the feature and the category, as well as calculate the feature’s correlation with Pearson’s correlation coefficient. Hence, the maximum correlation and minimum redundancy features are used to establish the optimal feature subset. And the feature’s objective function regarding the correlation and redundancy is traversed in the subset based on the greedy algorithm. Lastly, the features sorted in the subset are determined based on their priority.

Given a sample feature set $S=\{x_{1},x_{2},\cdots ,x_{n}\}$ and a sample class *c*, the mRMR method seeks the optimal features of the samples with maximal relevance *D*(*S*,*c*) and minimal redundancy *R*(*S*). Therefore, the features’ subset objective function is

(2) $$\underset{S}{\max}D(S,c)-R(S)$$

with

(3) $$D(S,c)=\frac{1}{\left|S\right|}\sum_{x_{i}\in S}I\left(x_{i};c\right),$$

(4) $$R(S)=\frac{1}{{\left|S\right|}^{2}}\sum_{x_{i},x_{j}\in S}I\left(x_{i},x_{j}\right),$$

where D represents the relevance between the sample feature set *S* and the sample class *c*, which is the mean of all mutual information between each feature $x_{i}$ and class *c*, and R represents the redundancy of all features in *S*, which is the mean of all mutual information between feature $x_{i}$ and feature $x_{j}$. We refer readers to [58] for more details about mRMR.

### 3.3. Stage 3: Activity Classification

This study tests several types of classification algorithms, including three major categories: traditional machine learning, deep learning, and transfer learning. Each model completes hyperparameter optimization based on the GS and RS methods, as well as performing 10 cycles of k-fold cross validation to find the result with the highest accuracy and effectiveness, which ensures that we can find the best human activity classifier.

**Figure 7:**
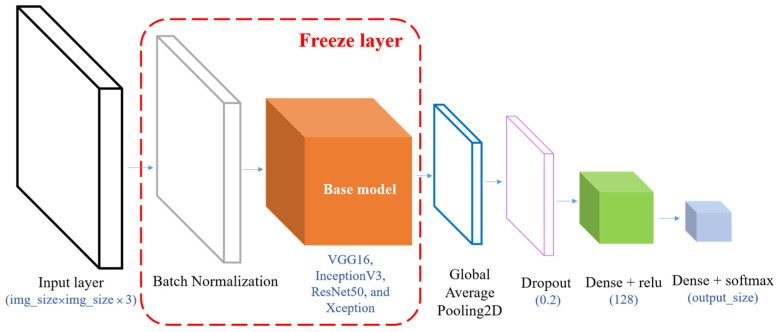
Neural network architecture of transfer learning.

In total, 11 types of classifiers based on traditional machine learning methods are explored, namely linear SVM, poly SVM, KNN, LDA, HMM, DT, RF, AdaBoost, XGboost, LightGBM, and Catboost. Table 3 describes the hyperparameters and optimization methods used in traditional machine learning algorithms. Moreover, five neural networks of DNN, BPNN, CNN, and CapsNET based on the concept of deep learning are developed to complete action classification. Table 4 depicts the proposed model architecture and related hyperparameter optimization methods used to create deep learning algorithms. Since it may be difficult to collect enough training data, we try to change the input data type to represent an input feature that fits the input of the powerful model. Accordingly, the grayscale image is converted into an RGB image, and its size is larger than 71 × 71 based on Keras documentation. The models all stand out in the ImageNet large-scale visual recognition challenge (ILSVRC) large-scale comparison, such as VGG16, InceptionV3, ResNet50, and Xception. Data migration is performed, extending from the well-trained source domain to the target training domain, based on the fine-tuning training method used to realize transfer learning and improve the model’s accuracy. Figure 7 presents the model’s architecture based on transfer learning performed in this study.

### 3.4. Stage 4: Data Generation

To alleviate the impact of having insufficient amount of existing training data, efficient data augmentation can be applied to improve accuracy and generalization performance. However, standard data augmentation can only produce surrogate data with limited confidence. In order to increase the breadth of the data, this study designs and trains several types of generative models used to complete data augmentation. Furthermore, we load the newly generated data onto the highest accuracy classifier to find the generator with the highest reliability, which can be used as the input model of the wrong sensor for the purpose of data correction. Table 5 summarizes the proposed generative model’s architecture. The following paragraphs include descriptions of the various types of generative models:

(1). Autoencoder (AE)

The autoencoder is often used to reduce noise and improve the quality of signal or image processing. With its powerful and flexible methods, it is suitable for use in both linear and non-linear datasets. AE is divided into the encoder and decoder. Firstly, the encoder extracts important features from the input data to reduce the data dimension and complete data compression. Then, the decoder reconstructs the data and generates new output data to ensure that the newly output data can express the same meaning as the input data.

(2). Convolutional Variational Autoencoder (CVAE)

Based on the CNN and variational autoencoder (VAE), CVAE creates a variety of continuous–discrete combinations by adding conditions to the encoding process to make the output follow a Gaussian distribution. CVAE extracts important features from the input data through the convolutional layer and filters unimportant features via the pooling layer to improve the model’s recognition capability in terms of capturing local features and effectively preventing the distortion of the generated data.

(3). Generative Adversarial Network (GAN)

GAN is a sample generative technique, the output of which resembles the input data distribution. It consists of two neural network models, namely a generator, which is responsible for generating data, and a discriminator, which distinguishes between the input and original data. A generator generates new samples by adding random noise to the original data and feeding it into a discriminator along with the original sample. A discriminator then finds the difference between the two samples by estimating the probability distribution of the new and original sample to further determine the fake new data. We note that the generator and the discriminator optimize each other’s performance in competition.

(4). Adversarial Autoencoders (AAE)

Integrating AE and GAN, the AAE generator consists of an encoder and decoder, which are different from those of GAN. After the encoder compresses the input data, a latent variable will be generated. Decoder will then try to decompress it and generate new fake data. Meanwhile, the discriminator will use latent variable as the input data through continuous learning to identify the credibility of its source data.

(5). Deep Convolutional Generative Adversarial Network (DCGAN)

Traditional GAN is mostly composed of fully connected layers, which cause its inability to express local features and poor resolution. DCGAN, which consists of CNN and GAN, effectively solves this problem. Using the powerful feature extraction ability of CNN, the learning effect of a generator can be improved. In addition, each neural layer has been standardized via batch normalization (BN) to reduce the variability between samples, which further stabilizes the model’s training process and reduces training problems caused by poor initialization.

(6). Conditional GAN (CGAN)

To solve the problem of GAN being unable to generate the specified category of data, CGAN passes the data containing the actual label to the network to improve the generator’s control over the output data and ensure that the generator and discriminator are only trained using the corresponding label to ensure that the convergence speed and performance of the model are effectively improved.

## 4. System Evaluation

In order to evaluate the proposed system, 100 pieces of activity data are captured from each activity performed in four activity categories (i.e., standing, sitting, walking, and running) via the developed sensor nodes. The activities are conducted by an average-sized young woman (average medium frame and an average height of 5′5″ feet). The data sampling of each category considers the period of sustaining the current action and the transition period from the current action to the new action. Thus, we capture a total of 400 pieces of data, with 80% of the data being the training data and 20% being the testing data. Then, the activity classification is executed based on the above classification model. We denote 10% of the training data as the verification data to ensure the model’s effectiveness. We notice that the k-fold cross validation is performed ten times and the average accuracy is used as the evaluation standard for each classifier. The computer hardware environment is equipped with a 3.7-gigahertz Intel Core i7 processor, 64 GB RAM, and an NVIDIA 2080 graphics card. The software environment is Tensorflow for Python, C, and C Sharp.

### 4.1. Singal Processing

**Figure 8:**
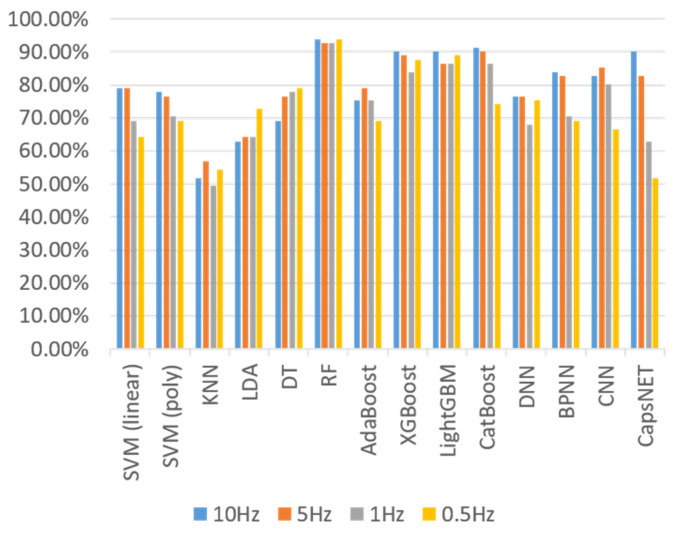
Accuracy results of each classifier at different sampling rates.

As the sampling rate decreases, the number of data features is reduced, which cuts down the model’s size and complexity, but may degrade the model’s effectiveness and classification accuracy. Thus, we perform down-sampling processing on the sequence of the original 10-hertz sampling rate (e.g., 5 Hz, 1 Hz, and 0.5 Hz) to find an appropriate sampling rate. Figure 8 shows the accuracy of directly feeding the sequence into various classifiers by varying the sampling frequency. The results show that a sampling rate of 10 Hz is generally a sensible setting among all classifiers.

### 4.2. Feature Engineering

**Figure 9:**
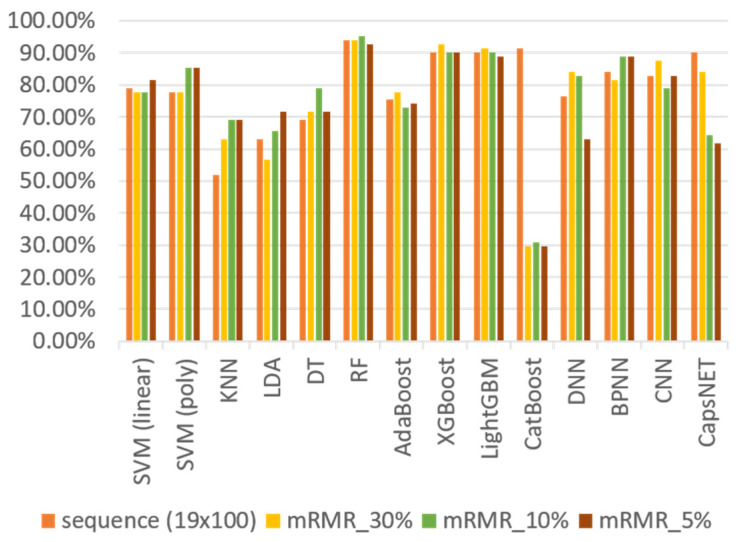
Accuracy results of each classifier when the input is the sequence.

To reduce the training time and prevent overfitting due to the influence of unimportant features, the mRMR method is applied to screen the features of the sequential input. Accordingly, the *k* features with the highest correlation and the least redundancy in the category are selected, while the selected features are fed into the classification model to perform training. Figure 9 shows the classification results without feature engineering and those determined via the mRMR method.

**Figure 10:**
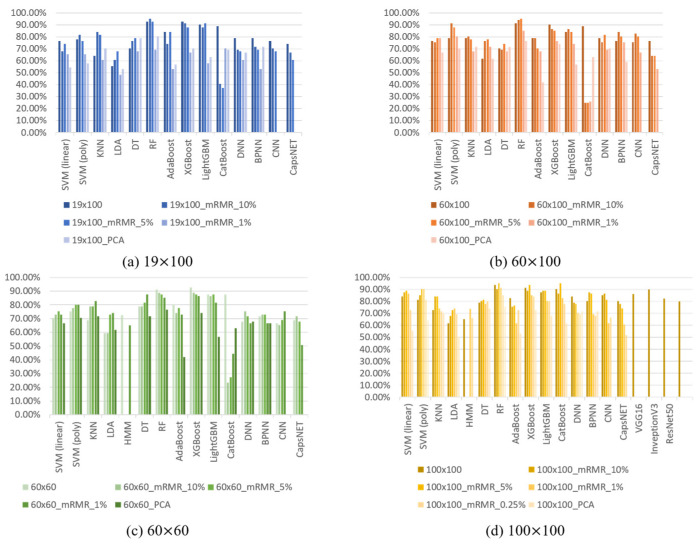
Accuracy results for each classifier when the inputs are images of various sizes.

Instead of using the sequential input, we implement the classification method of with input images at sizes of 19 × 100, 60 × 100, 60 × 60, and 100 × 100 via nearest neighbor interpolation (as described in Section 3.2) and reduce the number of excessive features in the image via the PCA and mRMR methods. Figure 10 shows the results of feeding the input images into the classification model after performing feature engineering.

Considering the traditional machine learning classifiers, Figure 9 and Figure 10 show that regardless of the feature processing method applied, the classification results of RF and XGBoost are generally the best options. Table 6 lists the accuracy results of RF and XGBoost with the original features and those determined via the feature engineering methods. The results show that when the image size is 100 × 100 and we select the best features with 5% of the original feature quantity based on using mRMR as the feature input, we can find the best classification results. The accuracies of RF and XGBoost via feature engineering are 95.06% and 93.83%, respectively, increasing the accuracies by 1.31% and 2.70%, respectively. We notice that although the accuracy of RF is higher than that of XGBoost, RF has the characteristics of being better at classifying known data but performing poorly on unknown data. Therefore, to ensure that the developed classifier has a wider availability in the real-time field, a large amount of user training data may be required. However, this approach may not only cause inconvenience to the subjects, but also lead to the possibility of overfitting the model by collecting too many similar data. Therefore, we propose a variety of generators to generate training data and load the newly generated data into the previously pre-trained RF and XGBoost classifiers to determine the desired classifier with the highest generalization capability.

### 4.3. Data Augmentation

**Figure 11:**
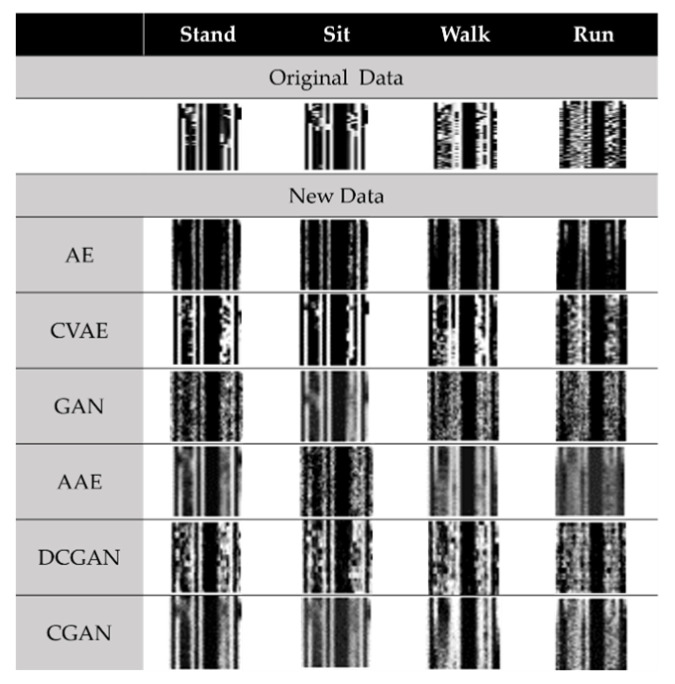
Generating new data for each category based on a generative model.

As mentioned in Section 4.2, feature engineering based on an image size of 100 × 100 and an important feature set based on mRMR by selecting 5% of the original feature numbers leads to the best classification performance. Therefore, new images with a size of 100 × 100 are generated to perform data augmentation. Meanwhile, a low-random noise is appropriately added to improve the noise resistance and generalization of the classification model. The action data containing the transition period extending from the current action to the new action are fed into the generative model according to each activity category. Each generator will generate 100 pieces of data for each category, equaling 400 new pieces of data in total. Figure 11 shows a typical run of generating new data. All six generators will generate a total of 2400 pieces of new data.

Subsequently, we combine all newly generated data with the training data in the original dataset to create a new training dataset with a total of 2720 pieces of data. As a result of the inputting of the training data into the previously pre-trained RF and XGBoost classifiers and their further re-training, Table 7 describes the accuracy results of the classifiers after data augmentation. The results show that RF may cause unsatisfactory model classification results due to the newly added fake data. This result occurs because of the fact that RF uses bagging and randomly extracts *k* features of a classification subset as decision-making factors. Thus, when we load an image with input features of 100 × 100, the accuracy of the model is less susceptible to the newly generated data due to the high resolution of the newly generated data and the preservation of the correlation between the signals.

### 4.4. Classifier and Generator

To find the most believable generator for newly generated data, we load the fake data into the un-retrained RF and XGBoost classification models. Table 8 lists the accuracy results derived by loading the data generated via each generative model into the pre-trained model. Referring to the results, in the both the RF and XGBoost models, the data generated via CVAE have the highest correct classification accuracy. We observe that XGBoost can even classify 80.75% of the new data, which results in the highest classification accuracy, implying that XGBoost may have a better performance than RF in terms of classifying unknown data.

### 4.5. Discussion

Comparing the existing HAR systems to an IMU node, the proposed system simulates the real-world environment as much as possible, which does not need to regulate the subject to complete the specified action within the specified time and limit of its action trajectory. Compared to existing AI-based HAR studies (e.g., SVM [19,26], DT [19], KNN [26], RF [26], HMM [27], AdaBoost [28], LSTM [36,39], and CNN [37,38,39]), we implement up to 19 classifiers and 6 generators and combine the two hyperparameter optimization methods of GS and RS to find the most suitable model. At the same time, we use feature engineering with two different concepts (i.e., selecting a subset of features with the most correlation with the output and the least correlation among these corresponding features) to reduce the feature dimension, find the ideal input features, further reduce the size of the classifier, and improve the time and accuracy of classification operations, providing a comprehensive study.

Based on the proposed system architecture and design, the open neural network exchange [59] format is applied to represent the classifier and generator models, being written in Python programming language during pre-training. Afterwards, the proposed system integrates the model into the UI written via C Sharp programming through the ML.NET framework [60] and combines the experimental results with development environments in programming to further realize the real-time HAR system.

For data pre-processing, the proposed system improves the generalization of the classification models and noise resistance through the proposed data augmentation strategy. Consequently, the resolution of the generative model required to perform detail generation is utilized through converting the inertial serial numbers into an image method to generate the most similar pieces of data and correct the inappropriate part of the data.

For feature engineering, the data generated via the proposed method have a relatively ideal classification accuracy as the input feature. We try various image sizes and combine PCA and mRMR with two feature engineering methods used to perform feature screening. The results show that when the image size is 100 × 100 and 5% of the original feature number is selected using the mRMR method to be the input feature of the classifier, the classification accuracy is the highest. Although we only have training data for one subject, we have placed the IMU node in the most suitable position, which allows us to capture the most accurate inertial signal. At the same time, we use the Madgwick filter to eliminate drift and errors caused by fast movements. If the node is worn by a user who is different in height and weight to the subject, the vibration will be similar, despite the different size of the inertial signal. Therefore, combined with the feature engineering method described earlier, the input features of the user will be similar to those of the subject, which means that the proposed system have good versatility.

For data generation, since we apply mRMR to select the features of the newly generated data, the number of features in the input classifier becomes 500 (i.e., 5%). Although we find the optimal subset of features based on the original dataset, we cannot guarantee that the selected features, according to the subset found in the newly generated dataset, maintain maximum correlation and minimum redundancy. At the same time, due to the reduction in a large number of features, the correlation between features may be reduced, which leads to performance degradation after using the mRMR algorithm. In contrast, XGBoost is effectively improved, regardless of whether it performs feature engineering.

In summary, based on the experimental results of the classification performance, the re-trained XGBoost and CVAE models are used as the final classifier and the final generator, respectively, with classification accuracy of 96.03%. While the accuracy of the classified actions is as expected, the proposed system can only classify the actions with large variations, which may limit its use in the field. Therefore, to be more in line with the real-world environment, we are planning to define actions with high classification complexity as future goals.

## 5. Conclusions

In this work, we propose a novel real-time human activity recognition system. The proposed system consists of four parts: data sampling, data pre-processing, a classifier, and a generator. To screen features and improve the model’s generalization, we preserve the correlation between multiple inertial signals by converting the sequence into images and integrating the mRMR method and the data augmentation strategy such that the IMU activity can still be identified, even with limited training data, inertial signal drift, and noise.

Based on models such as the traditional machine learning, deep learning, and transfer learning models, experimental results show that the accuracy levels of the RF and XGBoost models are generally greater under various feature engineering methods, especially when the image size is 100 × 100 pixels and the important features of 5% of the original features are selected via the mRMR method. However, after adding the unknown data, XGBoost has a better accuracy of 96.03%, while the accuracy of RF drops to 91.36%. Moreover, the newly generated data are fed into the classifier and combined with the aforementioned feature engineering to evaluate the best generator. Based on the experimental results, the data generated via CVAE are classified with the highest accuracy (80.75%), even in XGBoost without re-training, which implies that this CVAE generator has the highest effectiveness, while the generalization ability of XGBoost is better than that of RF. Therefore, the proposed system selects the pre-trained XGBoost and CVAE as the classifier and generator, respectively, which can be further integrated into the visual interface to complete the real-time HAR system. In a future work, we plan to collect action data related to diverse human activities, accomplish more complex HAR with lower system resources, improve the operational performance and efficiency, and improve the classification accuracy.

## Figures and Tables

**Table 1:** The comparison between different sensory methods and generative models.

Works	IMU Signals	Characteristics	Limitations
SensoryGAN [47]	Acc	Apply generative adversarial network to generate sensor data	Use corresponding generative models for individual actions
X. Zhang [48]	Acc Gyro Mag	Use the semi-supervised GAN	Control classification results using custom parameters
A. Mathur [49]	Acc Gyro	Incorporate the heterogeneity of different sensors to enrich the training set	The diversity of synthetic data is limited and not guaranteed.
E. Soleimani [50]	Acc	Realize the Transfer Learning and use GAN to generate source domain data from the target domain	Limited diversity of newly generated data
The Purposed System	Acc Gyro Mag	Enrich the training set via data synthesis based on generative models such as AE or GAN	Train the network with limited data

**Table 2:** The comparison of the specifications of the two most common nine-axis IMUs.

Product	Spec	ADC	Notes
BNO055 [54]	±125°/s to ±2000°/s	16 bits	gyros
	±2 g, ±4 g, ±8 g, ±16 g	14 bits	accel
	±1300 µT (*x*-, *y*-axis); ±2500 µT (*z*-axis)	0.3 µT	mag
MPU9150 [55] (the IMU used in this work)	±250, ±500, ±1000, ±2000°/s	16 bits	gyros
	±2 g, ±4 g, ±8 g, ±16 g	16 bits	accel
	±1200 µT	13 bits (0.3 µT per LSB)	mag

**Table 3:** Hyperparameters and the optimization of traditional machine learning methods.

Machine Learning	Hyperparameters	Definition	Search Range	Method
Linear SVM	Cost (c)	the penalty parameter	1 × 10^−5^~1 × 10^4^	GS
Poly SVM	Cost (c)	the penalty parameter	1 × 10^−5^~1 × 10^4^	GS
	Degree	the polynomial degree used to find the hyperplane required to split the data	2~6	
	Gamma	the extent to which the influence of a single training example reaches	0.1~100	
KNN	K	the K-Neighbors closest to the new data after calculating the distance	[3, 5, 7, 9, 11]	GS
LDA	-	-	-	-
HMM	-	-	-	-
DT	max_depth	the maximum depth of the tree	1~100	GS
	min_samples_split	the minimum number of samples required to split an internal node	1~40	RS
	min_samples_leaf	the minimum number of samples required at a leaf node	1~20	
RF	n_estimators	the number of trees present in the forest	100~1000	RS
	max_depth	the maximum depth of the tree	10~100	
	min_samples_leaf	the minimum number of samples required to split an internal node	1~20	
	min_samples_split	the minimum number of samples required at a leaf node	1~10	
AdaBoost	n_estimators	the number of base estimators or weak learners	50~100	GS
	learning_rate	shrinking the contribution of each classifier	1 × 10^−3^~5 × 10^−1^	RS
XGBoost	max_depth	the maximum depth of the tree	3~10	GS
	min_child_weight	the minimum sum of the instance weight needed in a child	5~8	
	eta (learning_rate)	The step size shrinkage used in the update step to prevent overfitting	1 × 10^−2^~3 × 10^−1^	RS
LightGBM	max_depth	limit the tree’s depth	3~12	GS
	num_leaves	control the complexity of the tree model	20~100	RS
	min_data_in_leaf	prevent overfitting in a leaf-wise tree	200~1000	
CatBoost	iterations	the maximum number of trees that can be built	[10, 100, 150, 200, 250]	GS
	depth	the depth of the tree	[2, 4, 6, 8]	
	learning_rate	the rate at which the model weights are updated after working through each batch of training examples	0.03~0.1	RS
	l2_leaf_reg	the coefficient for the L2 regularization term of the cost function	0.2~3.0	

**Table 4:** Neural network architecture of deep learning models.

Deep Learning	Hyperparameters	Optimization	Layer Type	Layer Information
DNN	learning_rate (LR): 1 × 10^−6^ batch_size: 2 epochs: 300	LR tuning from RS in the range of [1 × 10^−7^~5 × 10^−5^]	Fully Connected and Dropout [input]	input size: img_size × img_size, hidden units: 256, activation: relu, dropout: 0.4
			Fully Connected [hidden]	the number of layers: 4, hidden units: [16, 32, 64, 128], activation: relu
			Fully Connected [output]	hidden units: output_size, activation: relu
BPNN	learning_rate (LR): 1 × 10^−5^ batch_size: 2 epochs: 500	LR tuning from RS in the range of [5 × 10^−5^~3 × 10^−4^]	Dropout and Fully Connected [input]	input size: img_size × img_size, hidden units: 512, activation: relu, dropout: 0.5
			Dropout and Fully Connected [hidden]	hidden units: 128, activation: relu, dropout: 0.5
			Dropout and Fully Connected [output]	hidden units: output_size, activation: softmax, dropout: 0.5
CNN	learning_rate (LR): 2 × 10^−6^ batch_size: 2 epochs: 300	LR tuning from RS in the range of [1 × 10^−7^~5 × 10^−5^]	Conv2D and maxPool2D [input]	input size: [img_size, img_size, 1], kernels: [5 × 5], filters: 32, pool_size: [2 × 2], activation: relu
			Conv2D and maxPool2D	kernels: [5 × 5], filters: 64, pool_size: [2 × 2], activation: relu
			Dropout and Flatten	hidden units: 128, activation: relu, dropout: 0.5
			Fully Connected [output]	hidden units: output_size, activation: softmax
CapsNET	learning_rate (LR): 1 × 10^−4^ decay_rate: 1 × 10^−6^ batch_size: 4 epochs: 25	LR tuning from RS in the range of [2 × 10^−4^~1 × 10^−3^]	Conv2D, BN, and MaxPool2D [input]	input size: [img_size, img_size, 1], kernels: [2 × 2], filters: 32, pool_size: [2 × 2], activation: relu
			Conv2D, BN, and MaxPool2D	kernels: [2 × 2], filters: 64, pool_size: [2 × 2], activation: relu
			Conv2D, BN, and MaxPool2D	kernels: [2 × 2], filters: 128, pool_size: [2 × 2], activation: relu
			PrimaryCap	kernels: [2 × 2], dim_capsule: 4, n_channels: 16, strides: 1
			CapsuleLayer	num_capsule: output_size, dim_capsule: 4, num_routing: 3
			Dropout and Flatten	dropout: 0.8
			Fully Connected [output]	hidden units: output_size, activation: softmax

**Table 5:** Neural network architecture of generative model.

Generative Model	Neural Network		Layer Type	Layer Information
AE	Encoder		Fully Connected [input]	input size: img_size × img_size, hidden units: 128, activation: sigmoid
			Fully Connected [output]	hidden units: 64, activation: sigmoid
	Decoder		Fully Connected [input]	input size: 64, hidden units: 128, activation: sigmoid
			Fully Connected [output]	hidden units: img_size × img_size, activation: sigmoid
CVAE	Encoder		Conv2D and maxPool2D [input]	input size: [img_size, img_size, 1], kernels: [3 × 3], filters: 64, pool_size: [2 × 2], activation: relu
			Conv2D and maxPool2D	kernels: [3 × 3], filters: 128, pool_size: [2 × 2], activation: relu
			Flatten and Fully Connected [output]	hidden units: latent_dim
	Decoder		Fully Connected [input]	input size: [latent_dim], hidden units: ${\left(\frac{\mathrm{img}\_\mathrm{size}}{4}\right)}^{2}$ × 0.2564, activation: relu
			Deconv2D and UnmaxPool2D	kernels: [3 × 3], filters: 128, pool_size: [2 × 2], activation: relu
			Deconv2D and UnmaxPool2D	kernels: [3 × 3], filters: 64, pool_size: [2 × 2], activation: relu
			Deconv2D and UnmaxPool2D [output]	kernels: [3 × 3], filters: 1, pool_size: [1 × 1]
GAN	Generator		Fully Connected and Dropout [input]	input size: img_size × img_size, hidden units: 128, activation: leakyrelu, dropout: 0.2
			Fully Connected [output]	hidden units: img_size × img_size, activation: tanh
	Discriminator		Fully Connected [input]	input size: img_size × img_size, hidden units: 128, activation: leakyrelu
			Fully Connected [output]	hidden units: 1, activation: [relu, sigmoid]
AAE	Gene-rator	Encoder	Flatten [input]	input size: [img_size, img_size, 1]
			Fully Connected [hidden]	the number of layers: 2, hidden units: [512, 512], activation: leakyrelu
			Fully Connected [output]	the number of layers: 2, hidden units: [latent_dim, latent_dim]
		Decoder	Fully Connected [input]	input size: [latent_dim], activation: leakyrelu
			Fully Connected [hidden]	hidden units: 512, activation: leakyrelu
			Fully Connected [output]	hidden units: img_size × img_size, activation: tanh
	Discriminator		Fully Connected [input]	input size: [latent_dim], hidden units: 512, activation: leakyrelu
			Fully Connected [hidden]	hidden units: 256, activation: leakyrelu
			Fully Connected [output]	hidden units: 1, activation: sigmoid
DCGAN	Generator		Fully Connected [input]	input size: [g_dim]
			Deconv2D and BN	kernels: [5 × 5], filters: 32, activation: leakyrelu
			Deconv2D and BN	kernels: [5 × 5], filters: 16, activation: relu
			Deconv2D and BN [output]	kernels: [5 × 5], filters: 1, activation: tanh
	Discriminator		Conv2D and BN [input]	input size: [img_size, img_size, 1], kernels: [5 × 5], filters: 32, activation: relu
			Conv2D and BN	kernels: [5 × 5], filters: 64, activation: relu
			Fully Connected [output]	hidden units: 1, activation: sigmoid
CGAN	Generator		Fully Connected and BN [input]	input size: [latent_dim], hidden units: 64, activation: leakyrelu
			Fully Connected and BN [hidden]	the number of layers: 2, hidden units: [128, 256], activation: leakyrelu
			Fully Connected [output]	hidden units: img_size × img_size, activation: tanh
	Discriminator		Fully Connected [input]	input size: img_size × img_size, hidden units: 64, activation: leakyrelu
			Fully Connected [hidden]	the number of layers: 2, hidden units: [128, 256], activation: leakyrelu
			Fully Connected [output]	hidden units: 1, activation: sigmoid

**Table 6:** Best accuracy results in RF and XGBoost determined via various feature engineering methods.

	Size	Original Feature		FE			
		RF	XGBoost	RF		XGBoost	
				Acc	The Best Method	Acc	The Best Method
Sequence	19 × 100	93.83%	90.12%	95.06%	mRMR_10%	92.59%	mRMR_30%
Image	19 × 100	92.59%	92.59%	95.06%	mRMR_10%	91.36%	mRMR_10%
	60 × 100	91.36%	90.12%	95.06%	mRMR_5%	86.42%	mRMR_10%
	60 × 60	91.36%	92.59%	88.89%	mRMR_10%	88.89%	mRMR_10%
	100 × 100	93.83%	91.36%	95.06%	mRMR_5%	93.83%	mRMR_5%

**Table 7:** Accuracy results of RF and XGBoost after data augmentation.

	Original Feature		FE—mRMR_5%	
	RF	XGBoost	RF	XGBoost
Original	93.83%	91.36%	95.06%	93.83%
Re-training	93.83%	95.06%	91.36%	96.03%

**Table 8:** The new data of each generator are loaded into the pre-trained RF and XGBoost models at a accuracy level.

	Classifier	RF (Pre-Trained)	XGBoost (Pre-Trained)
Generator			
AE		46.25%	25.00%
CVAE		59.00%	80.75%
GAN		51.50%	51.50%
AAE		50.25%	63.00%
DCGAN		44.25%	47.75%
CGAN		35.00%	41.25%

## Data Availability

The author working at the Department of Computer Science and Engineering, National Chung Hsing University, Taiwan, was the subject of the experiments. The author agreed to participate in this research study.
